# Evaluation of a new two-step frailty assessment of head and neck patients in a prospective cohort

**DOI:** 10.1007/s00405-024-08651-8

**Published:** 2024-04-23

**Authors:** Beniamino Vincenzoni Padovan, M. A. J. Bijl, J. A. Langendijk, H. P. van der Laan, B. A. C. Van Dijk, S. Festen, G. B. Halmos

**Affiliations:** 1 Department of Otorhinolaryngology/Head and Neck Surgery, University of Groningen, University Medical Center Groningen, Groningen, The Netherlands; 2 Department of Radiation Oncology, University of Groningen, University Medical Center Groningen, Groningen, The Netherlands; 3 Department of Research and Development, Netherlands Comprehensive Cancer Organisation (IKNL), Utrecht, The Netherlands; 4 Department of Epidemiology, University of Groningen, University Medical Center Groningen, Groningen, The Netherlands; 5 University Medical Center Groningen, University Medical Center for Geriatric Medicine, Groningen, The Netherlands

**Keywords:** Head and neck oncology, Frailty, Geriatric screening, Adverse outcomes

## Abstract

**Purpose:**

Assessing frailty, in head and neck cancer (HNC) patients is key when choosing appropriate treatment. Optimal screening is challenging, as it should be feasible and should avoid over-referral for comprehensive geriatric assessment (CGA) This study aims to evaluate the association between geriatric assessment using a new two-step care pathway, referral to geriatrician and adverse outcomes.

**Methods:**

This institutional retrospective analysis on a prospective cohort analysed the multimodal geriatric assessment (GA) of newly diagnosed HNC patients. Uni- and multivariable logistic regression was performed to study the association between the screening tests, and referral to the geriatrician for complete geriatric screening, and adverse outcomes.

**Results:**

This study included 539 patients, of whom 276 were screened. Patients who underwent the GA, were significantly older and more often had advanced tumour stages compared to non-screened patients. Referral to the geriatrician was done for 30.8% of patients. Of the 130 patients who underwent surgery, 26/130 (20%) experienced clinically relevant postoperative complications. Of the 184 patients who underwent (radio)chemotherapy, 50/184 (27.2%) had clinically relevant treatment-related toxicity. Age, treatment intensity, polypharmacy and cognitive deficits, were independently associated with referral to geriatrician. A medium to high risk of malnutrition was independently associated with acute radiation induced toxicity and adverse outcomes in general.

**Conclusion:**

The current study showed a 30.8% referral rate for CGA by a geriatrician. Age, treatment intensity, cognitive deficits and polypharmacy were associated with higher rates of referral. Furthermore, nutritional status was found to be an important negative factor for adverse treatment outcomes, that requires attention.

**Supplementary Information:**

The online version contains supplementary material available at 10.1007/s00405-024-08651-8.

## Introduction

The median age of the global population is rising, and consequently the median age of all cancer patients as well as of patients with head and neck cancer (HNC) [1, 2]. Since HNC is the sixth most common malignancy worldwide and its incidence is rising, this trend has significant consequences [3]. Treatment decision in HNC is complex, balancing intensive curative options like surgery and (chemo)radiation versus impact on quality of life and functional status. Tailored treatment in HNC is complex and should take frailty, co-morbidities and treatment outcomes, like adverse events and functional outcome, into account [4]. The goal is to avoid both overtreatment of frail younger patients and undertreatment of fit older patients. This is challenging as the curative treatment regimens used are often intensive and multimodal [5]. It has been observed that HNC patients, regardless of their chronological age, are more frail due to multiple factors [1]. These include an unhealthy lifestyle with often lifelong alcohol or tobacco use, but also symptoms secondary to the tumour location and size such as malnutrition and loss of function [6–8]. Comorbidities associated with frailty include cardiopulmonary pathologies (COPD, atrial fibrillation, vascular disease, hypertension) and other systemic pathologies such as diabetes mellitus, kidney disease and cognitive impairment. Studies have shown that all these comorbidities have been associated with a worse prognosis as well as reduced quality of life post-treatment in HNC patients [1, 3].

Frailty is the depletion of reserves due to accumulation of deficits on different domains, like physical, functional, social and cognitive functioning [9]. It is a measure of biological ageing and a better predictor of the ability to endure stressful events compared to chronological age [10, 11]. The gold standard for the measurement of frailty is the comprehensive geriatric assessment (CGA), as it assesses all different geriatric domains such as polypharmacy, comorbidities, nutritional status, functional status, psychological status and social support [12]. CGA has been shown to reduce mortality and care dependence, but it is a time-consuming process, that requires sufficient resources and should be reserved for the group of patients most likely to benefit from it to limit patient burden [13–15]. Therefore, shorter screening tools have been developed, like the Geriatric 8 (G8) and the Groningen Frailty Indicator (GFI). G8 is a screening tool, which mainly focuses on the physical domain, containing questions related to nutrition, weight loss and comorbidities. In contrast, GFI is a 15-item questionnaire, including questions on physical, social and psychological domains. However, these have been found to have a lower prognostic value and a lower sensitivity compared to the full CGA; furthermore, also low specificity leading to over-referral for CGA [16]. A two-step method could provide the solution for adequate but time and energy efficient geriatric evaluation, i.e. an abbreviated geriatric screening first, followed by referral to a geriatrician for a CGA in case of frailty. The multidimensional character of the CGA can be maintained in the screening in the form of an abbreviated geriatric assessment. However, simplifying it to the essence: identifying problems that may lead to poor prognosis or poor treatment tolerance.

The aim of the current study was to investigate the effects the recently introduced geriatric care pathway in HNC patients, including a multidomain abbreviated geriatric screening. The new geriatric care pathway was designed to improve specificity of frailty screening: reducing unnecessary CGAs while identifying patients most likely to benefit. The study analysed the percentage of patients referred to the geriatrician for CGA and associations between assessment tools and referral. Furthermore, the possible association between patient characteristics, tumour characteristics and assessment tools; and adverse outcomes, including surgical complications and (chemo)radiotoxicity was studied.

## Materials and methods

### Study design and study population

The present study is a retrospective analysis, based on the OncoLifeS prospective, institutional database, an oncological data-bank approved by the Medical Ethical Committee and registered at the Dutch Trial Register (registration number NL7839) [17]. All patients who visited the outpatient clinic of the Department of Otorhinolaryngology/Head and Neck Surgery of the UMCG with a newly diagnosed or recurrent mucosal or non-mucosal, histology-confirmed head and neck malignancy between 1st January 2019 and 31st December 2020, were included. These included the skin (basal cell carcinoma, squamous cell carcinoma and melanoma) and non-skin tumours. The exclusion criteria consisted of no primary HNC, no malignancy in the inclusion period, patient died before treatment, or refused any treatment, if no data on treatment was available, and if patients were not fully screened during the first outpatient clinic appointment using the geriatric screening assessment.

The methodology of the study was performed according to the STROBE (Strengthening the Reporting of Observational Studies in Epidemiology) guidelines [18].

### Data collection

Patient data and oncological data were collected from the OncoLifeS database and supplemented with data from the electronic patient files. Data about (chemo)radiotherapy was collected from the prospective data registration program of the Department of Radiation Oncology of the UMCG Patients were divided into early stage (stage I and II) and advanced stage of disease (III and IV) classified according to the 8th edition of the TNM Classification of Malignant Tumours [19]. Treatment intensity was dichotomized into two groups: minor and major. Major intensity was defined as if surgery lasted more than 120 min or the radiation field included bilateral irradiation [20]. When a patient received multiple treatment modalities, the treatment intensity was considered major if the intensity of one of the single treatments was classified as major.

**Fig. 1:**
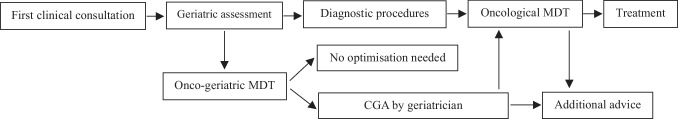
Clinical care pathway from first outpatient clinic contact to treatment

All patients were eligible for screening using a nurse-led geriatric assessment during the first appointment at the outpatient clinic (see Fig. 1), maximising the statistical power of the cohort and minimising selection bias The screening included multiple domains: somatic, psychological, functional and social. Every domain was assessed by one or more screening instruments. The somatic domain included the Malnutrition Universal Screening Tool (MUST), and polypharmacy, defined as the use of five or more medications on a daily basis. The psychological domain included delirium risk assessment (according to a nationwide guideline of the Dutch Safety Programme, VMSzorg, 2009) [21], the Mini Mental State Examination (MMSE) assessing cognitive impairments, the Patient Health Questionnaire-2 (PHQ-2) assessing signs of depression, and lastly an anamnestic evaluation of smoking and alcohol consumption (never/former or current user). The functional domain included the Katz Activities of Daily Living (ADL), and the Lawton Instrumental Activities of Daily Living (IADL), both assessing independence in daily activities of patients, the Time Up and Go Test (TUG) assessing patient mobility and an anamnestic evaluation of fall risk (at least one reported fall in the past 6 months). The socioenvironmental domain was assessed based on medical history, living situation (independent, independent with help or institutionalised) and the social network a patient had (limited or broad social network). Each assessment tool had a cut-off value validated through literature research (supplementary Table 1). Patients were discussed in an onco-geriatric multidisciplinary team (MDT) meeting, where the oncologic nurse presented the patient case, attended by a geriatrician, a head and neck surgeon, and sometimes a psychiatric nurse specialist, a radiation oncologist and a medical social worker. If the patient was considered to be frail based on the geriatric assessment and further geriatric evaluation seemed to be relevant, the patient was referred to the geriatrician for CGA. The CGA was performed before or after the oncological MDT and the advice of the geriatrician was taken into account when assessing the treatment plan (see Fig. 1).

### Outcome measures

The primary outcome measure was the percentage of patients referred to the geriatrician after undergoing the geriatric assessment. Uni- and multivariable analysis was performed to analyse the association between the individual geriatric instruments and referral for CGA.

The secondary outcome measures were the adverse events, divided into the surgical complications and toxicity related to (chemo)radiation. Clinically relevant surgical complications were defined as a grade 2 or higher according to the Clavien–Dindo classification [22]. All patients who received (chemo)radiotherapy both as a primary treatment as well as an adjuvant treatment were screened 12 weeks after the start of the therapy for (chemo)radiotoxicity. (Chemo)radiotoxicity grade 2 or higher, according to the Common Terminology Criteria for Adverse Events v4.0 (CTCAE v4.0) was deemed clinically relevant. The nine scored items were: dry mouth, dysgeusia, general pain, hoarseness, oral pain, mucositis, saliva, sore throat and weight loss. A last analysis was conducted including all patients, regardless of treatment modality. In this analysis, patients were classified as having adverse outcomes when either the Clavien–Dindo was grade 2 or higher, or the CTCAE was grade 2 or higher.

### Statistical analysis

The collected data was analysed using IBM SPSS Statistics v28.0 (Armonk, United States of America, 2021). Descriptive statistic was performed to compare the study population (patients who underwent geriatric screening during the first outpatient clinic appointment) with the cohort who was not screened. The data was presented as mean and standard deviation (SD), medians and interquartile range (IQR) or absolute number and percentage (%) based on the data characteristics. For normality and assumptions testing, the t-test was used for continuous data and the Pearson’s Chi square or Fisher’s exact test for categorical data. The association between geriatric assessment instruments, patient, tumour, and treatment characteristics and the outcome measures was analysed using a univariable logistic regression. Separate analyses were conducted for the single outcome measures. The outcome measures: referral to geriatrician, surgical complications, (chemo)radiotoxicity and overall adverse outcome were used as the dependent variables. The instruments of the geriatric assessment, patient, tumour, and treatment characteristics were considered as the independent variables. Odds ratio (ORs), 95% confidence intervals (95% CI) and *p*-values were calculated. Next, multivariable logistic regression analyses were performed with stepwise forward and backward selection to identify independent predictors of the outcome measures. Independent variables having a *p*-value < 0.10 were include in the multivariable analysis. The variables that did not fall out during the forward and backward selection were reported in the results section. Age was included regardless of the *p*-value. Pearson and Spearman correlation coefficients showed no collinearity between the multivariable model independent variables. A correlation of ≥ (−) 0.70 indicated collinearity between variables. A *p*-value of ≤ 0.05 indicated significance.

## Results

### Patient characteristics of the included and the excluded cohorts

**Fig. 2:**
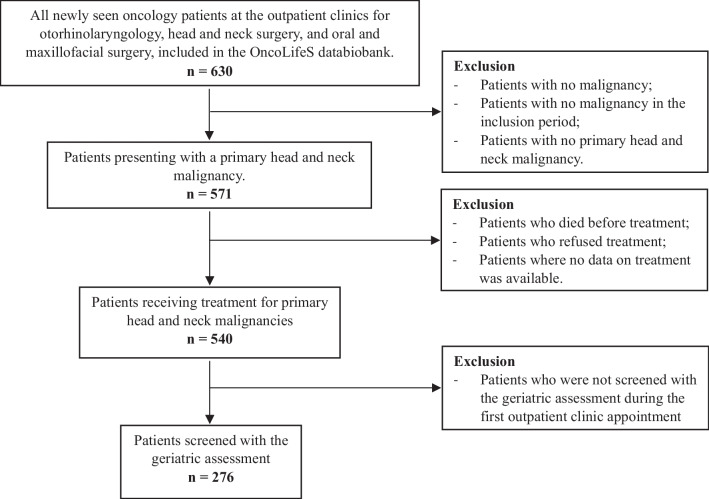
In- and exclusion flow diagram of the present study

During the inclusion period, 539 patients with HNC were treated. Two hundred seventy-six patients (51.2%) underwent geriatric assessment and were further analysed (Fig. 2 and Table 1). The mean age of this cohort was 73.1 years [SD ± 10.2], compared to 64.7 years [range 29.0–95.0] in the group who was not screened (*p* < 0.001), and patients were mainly male (*n* = 201, 72.8%). The screened group had more patients with polypharmacy (*p* < 0.001) and more patients with a history of alcohol abuse (*p* = 0.001). The tumour site mostly affected in the screened group was the skin, whereas in the non-screened group the lip and oral cavity were the most affected tumour site (*p* = 0.011). No significant differences were observed between the two groups in terms of gender, reason for referral (primary tumour or recurrence), histopathology, treatment intensity and history of smoking. An advanced tumour stage was more often seen in the screened group (*p* < 0.001) and fewer patients underwent surgery (*p* = 0.020). Treatment intensity was not significantly different between the screened and the unscreened group. In the screened cohort, most patients presented with a primary tumour (*n* = 258, 93.5%). The skin (*n* = 71, 25.7%), the lip and oral cavity (*n* = 60, 21.7%), the larynx (*n* = 51, 18.5%) and the oropharynx (*n* = 44, 15.9%) were the most common primary tumour sites. Squamous cell carcinoma (*n* = 233, 84.4%) was the most common histopathological diagnosis, with two-thirds of the patients presenting with an advanced tumour stage (*n* = 179, 64.9%). About half of the patients underwent surgery (*n* = 130, 47.1%) and more than half underwent treatment classified as major (*n* = 163, 59.1%).

**Table 1:** Patient and disease characteristics of the cohorts [*n* (%), unless specified otherwise]

Variable	Patients who underwent geriatric assessment *n* = 276	Patients who did not undergo geriatric assessment *n* = 263	*p*-value
Patient characteristics			
Age			
Mean ± SD, y	73.1 ± 10.2 y	64.7 ± 11.8 y	**< 0.001**
Median (range), y	73.0 (41.0–94.0)	66.0 (29.0–95.0)	
Sex			
Male	201 (72.8%)	178 (67.7%)	0.191
Female	75 (27.2%)	85 (32.3%)	
Intoxications			
Smoking			
Never or former	178 (64.5%)	124 (47.1%)	0.555
Current	83 (30.1%)	51 (19.4%)	
Unknown	15 (5.4%)	88 (33.5%)	
Drinking			
Never or former	131 (47.5%)	60 (22.8%)	**0.001**
Current	129 (46.7%)	112 (42.6%)	
Unknown	16 (5.8%)	91 (34.6%)	
Polypharmacy			
< 5 medications	111 (40.2%)	113 (43.0%)	**< 0.001**
≥ 5 medications	151 (54.7%)	63 (24.0%)	
Unknown	14 (5.0%)	87 (33.1%)	
Tumour characteristics			
Reason for referral			
Primary tumour	258 (93.5%)	250 (95.1%)	0.431
Recurrence	18 (6.5%)	13 (4.9%)	
Tumour site			
Skin	71 (25.7%)	37 (14.1%)	**0.011**
Lip, oral cavity	60 (21.7%)	76 (28.9%)	
Larynx	51 (18.5%)	61 (23.2%)	
Oropharynx	44 (15.9%)	41 (15.6%)	
Hypopharynx	14 (5.1%)	12 (4.6%)	
Other	23 (8.3%)	31 (11.8%)	
Unknown	13 (4.7%)	5 (1.9%)	
Stage of disease			
Early stage (0–II)	93 (33.7%)	140 (53.2%)	**< 0.001**
Advanced stage (III–IV)	179 (64.9%)	122 (46.4%)	
Not applicable	4 (1.5%)	0 (0.0%)	
Unknown	0 (0%)	1 (0.4%)	

Bold indicates statistical significance of the findings

### Primary outcome: referral to the geriatrician for comprehensive geriatric assessment

Out of the 276 patients who were screened, 85 (30.8%) were referred to the geriatrician for CGA. Univariable logistic regression showed that patients who were older of age (OR 1.03; 95% CI. 1.01–1.06), had an advanced tumour stage (OR 2.19; 95% CI. 1.22–3.95), underwent major treatment intensity (OR 2.01; 95% CI. 1.12–3.63), had polypharmacy (OR 3.07; 95% CI. 1.73–5.46), a declined cognition (OR 3.60; 95% CI. 1.68–7.71), restrictions in ADL (OR 3.95; 95% CI. 1.80–8.69), restrictions in IADL (OR 2.03; 95% CI. 1.09–3.78), and a prolonged TUG (OR 3.32; 95% CI. 1.38–8.00) were more likely to be referred to the geriatrician (Table 2). In the multivariable analysis older age (OR 1.06; 95% CI. 1.02–1.11), major treatment intensity (OR 2.77; 95% CI. 1.22–6.27), polypharmacy (OR 2.71; 95% CI. 1.29–5.70), and a declined cognition (OR 2.98; 95% CI. 1.27–6.97) were independently associated with referral to a geriatrician (*p* < 0.05) (Table 2).

**Table 2:** Association between geriatric assessment instruments, patient, tumour, and treatment characteristics and referral to the geriatrician (univariable and multivariable logistic regression)

Variable	Total N (%)	Not referred N (%)	Referred (%)	Univariable analysis^1^		Multivariable analysis^2^	
	*n* = 276	*n* = 190	*n* = 85	*p*-value	Odds ratio (95% CI)	*p*-value	Odds ratio (95% CI)
Age							
Mean ± SD (y)	73.1 ± 10.2 y	72.1 ± 10.4 y	75.2 ± 9.3 y	***p* = 0.021**	**1.03 (1.01–1.06)**	***p* = 0.003**	**1.06 (1.02–1.11)**
Sex							
Female Male Missing	75 (27.2%) 201 (72.8%) 0 (0.0%)	48 (25.3%) 142 (74.7%)	27 (31.8%) 58 (68.2%)	*p* = 0.264	1 0.73 (0.41–1.27)		
Intoxications							
Smoking							
Never or former Current Missing	178 (64.5%) 83 (30.1%) 15 (5.4%)	118 (62.1%) 59 (31.1%)	59 (69.4%) 22 (25.9%)	*p* = 0.477	1 0.81 (0.46–1.44)		
Drinking							
Never or former Current Missing	131 (47.5%) 129 (46.7%) 16 (5.8%)	86 (45.3%) 90 (47.4%)	44 (51.8%) 39 (45.9%)	*p* = 0.533	1 0.85 (0.50–1.43)		
Stage of disease							
Early stage (0–II) Advanced stage (III–IV) Missing	93 (33.7%) 179 (64.9%) 4 (1.5%)	73 (38.4%) 114 (60%)	19 (22.4%) 65 (76.5%)	***p* = 0.009**	**1** **2.19 (1.22–3.95)**		
Treatment intensity							
Minor Major Missing	92 (33.3%) 163 (59.1%) 21 (7.6%)	71 (37.4%) 104 (54.7%)	20 (23.5%) 59 (69.4%)	***p* = 0.020**	**1** **2.01 (1.12–3.63)**	***p* = 0.015**	**1** **2.77 (1.22–6.27)**
Somatic/physic							
Polypharmacy							
< 5 medications ≥ 5 medications Missing	111 (40.2%) 151 (54.7%) 14 (5.0%)	89 (46.8%) 88 (46.3%)	21 (24.7%) 63 (74.1%)	***p* = < 0.001**	**1** **3.07 (1.73–5.46)**	***p* = 0.009**	**1** **2.71 (1.29–5.70)**
MUST							
Low risk (< 1) Medium to high risk (≥ 1) Missing	135 (48.9%) 42 (15.2%) 99 (35.9%)	106 (55.8%) 31 (16.3%)	28 (32.9%) 11 (12.9%)	*p* = 0.472	1 1.34 (0.60–3.00)		
Psychological							
Delirium risk							
Low risk (< 1) High risk (≥ 1) Missing	112 (40.6%) 118 (42.8%) 46 (16.7%)	86 (45.3%) 89 (46.8%)	25 (29.4%) 29 (34.1%)	*p* = 0.715	1 1.12 (0.61–2.07)		
MMSE							
Normal cognition (> 24) Declined cognition (≤ 24) Missing	193 (69.9%) 34 (12.3%) 49 (17.8%)	154 (81.1%) 18 (9.5%)	38 (44.7%) 16 (18.8%)	***p* = < 0.001**	**1** **3.60 (1.68–7.71)**	***p* = 0.012**	**1** **2.98 (1.27–6.97)**
PHQ-2							
No depression (< 3) Depression (≥ 3) Missing	221 (80.1%) 30 (10.9%) 25 (9.1%)	146 (76.8%) 23 (12.1%)	74 (87.1%) 7 (8.2%)	*p* = 0.262	1 0.60 (0.25–1.46)		
Functional							
KATZ-ADL							
No restrictions (≤ 1) Restrictions (> 1) Missing	206 (74.6%) 30 (10.9%) 40 (14.5%)	159 (83.7%) 14 (7.4%)	46 (54.1%) 16 (18.8%)	***p* = < 0.001**	**1** **3.95 (1.80–8.69)**		
IADL							
No restrictions (> 6) Restrictions (≤ 6) Missing	111 (40.2%) 110 (39.9%) 55 (19.9%)	90 (47.4%) 74 (38.9%)	21 (24.7%) 35 (41.2%)	***p* = 0.026**	**1** **2.03 (1.09–3.78)**		
TUG							
Normal (< 12 s) Delayed (≥ 12 s) Missing	183 (66.3%) 24 (8.7%) 69 (25.0%)	144 (75.8%) 13 (6.8%)	37 (43.5%) 11 (12.9%)	***p* = 0.008**	**1** **3.32 (1.38–8.00)**		
Fall risk/History of falls							
No Yes Missing	173 (62.7%) 20 (7.2%) 83 (30.1%)	132 (69.5%) 13 (6.8%)	40 (47.1%) 7 (8.2%)	*p* = 0.252	1 1.78 (0.66–4.76)		
Social							
Living situation							
Independent/Independent with help Institutionalized Missing	260 (94.2%) 11 (4.0%) 5 (1.8%)	179 (94.2%) 6 (3.2%)	80 (94.1%) 5 (5.9%)	*p* = 0.315	1 1.87 (0.55–6.29)		
Social network							
Small Good Missing	33 (12.0%) 169 (61.2%) 74 (26.8%)	23 (12.1%) 130 (68.4%)	10 (11.8%) 38 (44.7%)	*p* = 0.346	1 1.49 (0.65–3.40)		

Bold indicates statistical significance of the findings

^1^Variables eligible for the forward and backward selection: age, stage of disease, treatment intensity, polypharmacy, MMSE, KATZ-ADL, IADL, TUG

^2^Variables included after forward and backward selection in the multivariable analysis and with a statistically relevant association

### Secondary outcomes

#### Association between geriatric assessment and overall adverse outcomes

In the study cohort 75 (28.5%) patients experienced clinically relevant (grade 2 or higher) adverse events after treatment. The univariable and multivariable analyses are described in Table 3. Multivariable analysis showed that a younger age (OR 0.94; 95% CI. 0.90- 0.99), advanced tumour stage (OR 3.72; 95% CI. 1.57–8.83) and a medium to high risk of malnutrition (OR 4.00; 95% CI. 1.47–10.88) were independently associated with the occurrence of adverse event (*p* < 0.05) (Table 3).

**Table 3:** Association between geriatric assessment instruments, patient, tumour, and treatment characteristics and adverse outcomes (univariable and multivariable logistic regression)

Variable	Total N (%)	No adverse outcome N (%)	Adverse outcome N (%)	Univariable analysis^1^		Multivariable analysis^2^	
	*n* = 263	*n* = 139	*n* = 75	*p*-value	Odds ratio (95% CI)	*p*-value	Odds ratio (95% CI)
Age							
Mean ± SD (y)	73.2 ± 10.1 y	74.3 ± 9.6 y	69.7 ± 9.7 y	***p* = 0.001**	**0.95 (0.92–0.98)**	***p* = 0.008**	**0.94 (0.90–0.99)**
Sex							
Female Male Missing	68 (25.9%) 195 (74.1%) 0 (0.0%)	33 (23.7%) 106 (76.3%)	23 (30.7%) 52 (69.3%)	*p* = 0.273	1 0.70 (0.38–1.32)		
Intoxications							
Smoking							
Never or former Current Missing	171 (65.0%) 77 (29.3%) 15 (5.7%)	94 (67.6%) 34 (24.5%)	43 (57.3%) 30 (40.0%)	***p* = 0.035**	**1** **1.93 (1.05–3.55)**		
Drinking							
Never or former Current Missing	126 (47.9%) 121 (46.0%) 16 (6.1%)	63 (45.3%) 64 (46.0%)	34 (45.3%) 38 (50.7%)	*p* = 0.746	1 1.10 (0.62–1.96)		
Tumour site							
Skin Other Missing	68 (25.9%) 182 (69.2%) 13 (4.9%)	38 (27.3%) 93 (66.9%)	10 (13.3%) 61 (81.3%)	***p* = 0.020**	**1** **2.49 (1.16–5.37)**		
Stage of disease							
Early stage (0-II) Advanced stage (III-IV) Missing	92 (35.0%) 168 (63.9%) 3 (1.1%)	65 (46.8%) 72 (51.8%)	13 (17.3%) 61 (81.3%)	***p* = < 0.001**	**1** **4.24 (2.13–8.41)**	***p* = 0.003**	**1** **3.72 (1.57–8.83)**
Treatment intensity							
Minor Major Missing	92 (35.0%) 163 (62.0%) 8 (3.0%)	53 (38.1%) 83 (59.7%)	8 (10.7%) 67 (89.3%)	***p* = < 0.001**	**1** **5.35 (2.38–12.02)**		
Referred to geriatrician							
No Yes Missing	180 (68.4%) 82 (31.2%) 1 (0.4%)	103 (74.1%) 35 (25.2%)	49 (65.3%) 26 (34.7%)	*p* = 0.153	1 1.56 (0.85–2.88)		
Somatic/physic							
Polypharmacy							
< 5 medications ≥ 5 medications Missing	106 (40.3%) 142 (54.0%) 15 (5.7%)	60 (43.2%) 71 (51.1%)	31 (41.3%) 39 (52.0%)	*p* = 0.800	1 1.08 (0.60–1.93)		
MUST							
Low risk (< 1) Medium to high risk (≥ 1) Missing	130 (49.4%) 37 (14.1%) 96 (36.5%)	81 (58.3%) 10 (7.2%)	32 (42.7%) 15 (20.0%)	***p* = 0.004**	**1** **3.80 (1.55–9.33)**	***p* = 0.007**	**1** **4.00 (1.47–10.88)**
Psychological							
Delirium risk							
Low risk (< 1) High risk (≥ 1) Missing	108 (41.1%) 110 (41.8%) 45 (17.1%)	55 (39.6%) 62 (44.6%)	32 (42.7%) 26 (34.7%)	*p* = 0.286	1 0.71 (0.38–1.33)		
MMSE							
Normal cognition (> 24) Declined cognition (≤ 24) Missing	182 (69.2%) 33 (12.5%) 48 (18.3%)	101 (72.7%) 16 (11.5%)	48 (64.0%) 8 (10.7%)	*p* = 0.913	1 1.05 (0.42–2.63)		
PHQ-2							
No depression (< 3) Depression (≥ 3) Missing	212 (80.6%) 26 (9.9%) 25 (9.5%)	113 (81.3%) 13 (9.4%)	65 (86.7%) 6 (8.0%)	*p* = 0.670	1 0.80 (0.29–2.21)		
Functional							
KATZ-ADL							
No restrictions (≤ 1) Restrictions (> 1) Missing	196 (74.5%) 27 (10.3%) 40 (84.8%)	108 (77.7%) 13 (9.4%)	54 (72.0%) 4 (5.3%)	*p* = 0.415	1 0.62 (0.19–1.98)		
IADL							
No restrictions (> 6) Restrictions (≤ 6) Missing	106 (40.3%) 104 (39.5%) 53 (20.2%)	62 (44.6%) 53 (38.1%)	32 (42.7%) 24 (32.0%)	*p* = 0.690	1 0.88 (0.46–1.67)		
TUG							
Normal (< 12 s) Delayed (≥ 12 s) Missing	174 (66.2%) 23 (8.7%) 66 (25.1%)	99 (71.2%) 9 (6.5%)	46 (61.3%) 7 (9.3%)	*p* = 0.335	1 1.67 (0.59–4.77)		
Fall risk/History of falls							
No Yes Missing	165 (62.7%) 18 (6.8%) 80 (30.4%)	93 (66.9%) 9 (6.5%)	45 (60.0%) 5 (6.7%)	*p* = 0.814	1 1.15 (0.36–3.63)		
Social							
Living situation							
Independent/Independent with help Institutionalized Missing	249 (94.7%) 10 (3.8%) 4 (1.5%)	134 (96.4%) 2 (1.4%)	72 (96.0%) 2 (2.7%)	*p* = 0.539	1 1.86 (0.26–13.49)		
Social network							
Small Good Missing	32 (12.2%) 158 (60.1%) 73 (27.8%)	17 (12.2%) 86 (61.9%)	10 (13.3%) 44 (58.7%)	*p* = 0.751	1 1.15 (0.49–2.72)		

Bold indicates statistical significance of the findings

^1^Variables eligible for the forward and backward selection: age, smoking, stage of disease, treatment intensity, MUST

^2^Variables included after forward and backward selection in the multivariable analysis and with a statistically relevant association

#### Association between geriatric assessment items and postoperative complications

Of the 130 patients who underwent surgery, 6 (4.6%) had a grade 1 complication, based on the Clavien–Dindo score and 20 (4.6%) had grade 2 or higher complications. Univariable and multivariable analyses are shown in supplementary Table 2. The multivariable analysis showed that major treatment intensity (OR 26.55; 95% CI. 3.11–226.29) and a lower delirium risk (OR 0.18; 95% CI. 0.05–0.65) were independently associated with postoperative complications (*p* < 0.05).

#### Association between geriatric assessment and (chemo)radio toxicity

Of the 184 patients who received (chemo)radiotherapy, 62 (33.7%) experienced grade 1 toxicity and 50 (27.2%) experienced grade 2 or higher toxicity. For 55 patients, no data was registered on the (chemo)radiotoxicity at 12 weeks. Univariable and multivariable analyses can be seen in supplementary Table 3. Multivariable analysis showed that a medium to high malnutrition score (OR 3.31; 95% CI. 1.08–10.21) and a younger age (OR 0.93; 95% CI. 0.88–0.99) were independently associated with the occurrence of acute radiation-induced toxicity (*p* < 0.05).

## Discussion

This study showed that 30.8% of HNC patients in a tertiary head and neck clinic were referred to a geriatrician for a CGA based on a nurse-led geriatric screening assessment. In comparison with other short single screening instruments, less patients needed to be referred, suggesting higher specificity and sensitivity of our care pathway. However, this percentage is still rather high, emphasizing the high prevalence of frailty in the HNC population. Polypharmacy, cognitive deficits, age and major treatment intensity were associated with CGA referral, suggesting these factors as main contributors to frailty. Furthermore, malnutrition was independently related to more adverse treatment related events, underlining the importance of screening for malnutrition and nutritional prehabilitation before treatment.

Our study population reflects the higher prevalence of HNC in older males. The fact that polypharmacy and alcohol abuse were seen more often in the screened cohort underlines that these factors can be regarded as predictors for frailty, especially in the HNC patients. Furthermore, it is also not surprising that in the screened cohort, patients had more often an advanced stage, therefore needing more aggressive or multimodal treatment. This can be explained by the more complex decision making process in this group; it is crucial to evaluate frailty to be able to weight survival against functional outcomes and post-treatment quality of life.

The decision to refer one third of the screened patients to the geriatrician for CGA was based on the combined outcome of the geriatric screening and the onco-geriatric MDT. Polypharmacy, cognitive deficits, age and major treatment intensity seem to be strongly associated with frailty, which explains why these four factors were predictive for referral. Previous studies have also found that cognitive deficits are associated with frailty. Clegg et al. [9] and Handforth [23] found that impaired cognition is associated with frailty. Other studies have also showed that impaired cognition is a predictor of adverse outcomes after treatment [24]. Polypharmacy has also been found in literature to be associated with not only frailty, but also postoperative complications, comorbidities, delirium risk, (chemo)radiotoxicity, mortality and a prolonged hospital stay when admitted [25, 26]. The association between age and referral to the geriatrician for CGA can be explained by the fact that although frailty is a different measure than chronological age, there is a very strong association between chronological age and frailty [9, 27]. However, this relation has been observed in a general population and seems to be less strong in the specific population of head and neck cancer, as shown earlier [1].

Furthermore, the multivariable analysis showed that a major treatment intensity was associated with referral to the geriatrician. This can be explained by the decision making during the onco-geriatric MDT, as patients with expected minor treatment (e.g. transoral laser surgery) were spared from time-consuming CGA. This reflects the importance of the present care pathway, namely, to refer for CGA only the patients that will benefit the most from it in terms of quality of life post-intervention. This decision is both based on the geriatric screening assessment as well as on the onco-geriatric MDT. Patients during the diagnostic workup need to undergo an extensive array of investigations which are time and energy consuming. The CGA is not a goal on its own but only a tool to help the decision making, and therefore should be reserved for a selected group.

The geriatric screening and two-step care pathway that have been analysed in the current study have identified 30.8% of the screened patients as possibly being frail and therefore needing a CGA. Comparing this with short screening tools such as the G8 or the Groningen Frailty Index (GFI); these short screening tools identified possible frailty often in more than half of the patients [28–30]. In a mixed cohort of cancer patients, G8 was impaired in 82% of the patients; however, this study included only patients above 70, which may explain the very high percentage [28]. In another study, evaluating the performance of GFI, almost 40% of the patients were frail [29]. This much lower percentage is very likely due to the different cohort, as the study by Drubbel et al. included patients from primary care, which is substantially different from a cancer population. This leads to a higher number of referrals to the geriatrician for CGA. Therefore, the new care pathway seems to lead to a decreased referral rate and therefore to a decrease in unnecessary CGAs performed by the geriatrician. Although the impact of the new care pathway on treatment choice and quality of life must be further analysed in future studies. Nevertheless, our study shows that abbreviated screening leads to less referrals for CGA, reducing not only patients’ burden but also spares unnecessary load of patients at the geriatric department, where more time remains for patients who really benefit from CGA.

Impaired KATZ-ADL and IADL performance were also significantly associated with referral to the geriatrician for CGA. This is in line with previous literature which showed that restrictions in the ADL have been associated not only with frailty but also with increased chance of adverse outcomes after treatment [31]. Also the TUG has been showed in the univariable analysis to be a significant predictor of referral to the geriatrician. Previous studies have found a correlation between frailty and a prolonged TUG, and this supports our results [32, 33]. TUG is measuring mobility and therefore it has a strong association with the KATZ-ADL and the ADL. This underlying association could explain why when analysing the data it could not be included in the multivariate analysis.

Multivariable analysis showed an association between adverse outcomes after treatment, regardless of modality and a higher risk of malnutrition, younger age and an advanced tumour stage. Malnutrition is an underdiagnosed and undertreated problem especially in the elderly population. It has been showed to be predictive for frailty, adverse outcomes in treatment and longer hospital stays [34, 35]. Furthermore it has also been showed that dietary counselling before and during radiotherapy significantly improves toxicity symptoms [36]. A previous study by Bras et al. found an association between malnutrition and adverse outcomes [37]. However, this was only for the intermediate risk group and not the high risk one. This surprising finding has been explained by a likely consequence of the pre-treatment dietary interventions in the high risk group. Based on these results, it can be assumed that nutritional intervention in the intermediate risk patients could be beneficial in preventing adverse outcomes after treatment. Our study further emphasizes the importance of recognizing malnutrition. Malnutrition can also be treated; however, specifically, in HNC, nutritional interventions can be challenging due to the limited time (as HNC is fast growing) and suboptimal patient compliance. Interventional studies should investigate the possibilities of nutritional prehabilitation and rehabilitation.

A younger age has been associated not only with adverse outcomes in general, but also with (chemo)radiotoxicity specifically. This can be explained by the fact that that patients above 70 years of age do not receive chemotherapy, following the guidelines of the Dutch Head and Neck Society, based on the meta-analysis of Pignon [38]. Therefore, younger patients have a higher chance of getting a more intensive treatment, leading to more adverse events. Another possible explanation is that older patients received a less aggressive treatment as a consequence of the decision during the MDT to adjust treatment intensity due to frailty. Previous studies have shown that in 28% of the cases, the outcome of the MDT was a modified treatment plan, often towards a less intense or even a palliative treatment [39, 40]. Next to this, the interventions (i.e. optimalisation), indicated by the geriatricians optimised the general status of the patients, leading to less adverse events. In the univariable analysis a major treatment intensity has also been showed to predict adverse treatment outcomes. This is also true for the multivariable analysis of the surgical adverse outcomes. This is in line with the findings of previously published studies [37, 41]. This literature data also supports the finding that advanced tumour stage is associated with a higher chance of experiencing adverse treatment outcomes.

Smoking and the tumour site have also been found, in the univariable analysis, to be associated with adverse outcomes. In line with these findings, smoking has been proven in previous literature to be a predictor of frailty and adverse treatment outcomes [7]. In our cohort, patients with skin tumours were less likely to experience adverse treatment outcomes. Skin tumours require a less complex and less physically straining treatment when comparing them with the same stage cancers at other sites in the head and neck area. This consequently leads to a lower chance of adverse treatment outcomes.

A lower delirium risk was associated with a higher chance of postoperative complications. This is a rather surprising finding and not in line with previous studies [25, 37]. Previous studies analysed a different care pathway and therefore are not directly comparable with our results. The present study analysed a pathway where optimisation and preventive measures were taken to optimise the health status of patients before they underwent treatment. Patients with a high delirium risk received delirium preventive measures (such as preventive rooming-in) or early detection and treatment of this condition. Furthermore, because by delirium-prone patients specific scores need to be regularly filled in by the nursing staff, this specific group of patients are checked upon more often during the day than a not delirium-prone patient. The optimisation and preventive measures together with the more intensive care could lead to a reduction of the incidence of adverse outcomes thanks to an early detection of these and an early. The influence of pre-habitation interventions has been seen earlier, in the context of malnutrition, as described above [37].

Future research should look at the impact of the geriatric screening on the treatment decision making, as well as the impact of treatment adjustment on the adverse outcomes and the survival. Furthermore a direct comparison between the current and the previous care pathway could bring more insights into the impact of the new care pathway in the patient care. Furthermore, randomized trials of pathway implementation in a multicentre setting can be performed in the future, where assessment of treatment modifications and the effect on different outcomes, including patient-reported outcomes can be investigated.

### Strengths and limitations

The analysed care pathway was conceived based on clinical experience and tested directly in a real-life situation on the final target group. This increases its clinical applicability. The choice of tests and cut-off scores for the geriatric screening was done using a strong literature basis for the validation. Outcome measures were quantified with objective scales (Claven-Dindo, CTACAE 4.0) which makes the results comparable and reproducible.

However, the present study has some limitations. A notable portion of the patients were not included in the analysis as they did not undergo the geriatric assessment at the fist outpatient clinic appointment. It is uncertain what the selection criteria for screening by the nurses at the first outpatient clinic appointment was. Logistic reasons (for example time pressure, understaffed clinics and lack of trained screeners at any given time) played a role in the decision not to screen a patient. This created a selection bias. Nurses often used ‘eyeballing’ to try and optimise the resources available and not screen what they considered ‘healthy’ or ‘not frail looking’ patients. This method has been proven to be less effective in estimating frailty in patients compared to evidence based scores [42–45]. This may lead to the study group not being fully representative of the head and neck cancer cohort. Furthermore, some of the exclusion criteria (such as patient died before treatment, or refused any treatment, missing data on treatment and missing key geriatric screening assessment data) may have also created bias.

Another limitation of the current study is the large amount of missing data. Due to the exclusion of a vast portion of patients due to incomplete or missing geriatric screening data, the subgroups during the analysis were relatively small. This may have led to a loss in statistical power. Lastly, the type of care pathway analysed in the current study may have interfered with the quality of the results. Patients after undergoing geriatric assessment underwent optimisation if necessary and the treatment plan was modified in some cases. This may have interfered with the analysis of the predictive value of the geriatric assessment tests and adverse treatment outcomes. This has been observed with the malnutrition and delirium risk scores in the current study.

Furthermore, the relatively limited sample size may also hamper the analysis, especially for the secondary outcome measures.

Finally, interpretation of the data for a different clinical practice can be difficult. The logistics of the geriatric screening was developed for our clinical institution specifically and translation to another oncology centre with different patient load could be challenging.

## Conclusions and clinical implication

The current study analyses assesses the clinical observations related to a recently implemented two-step care pathway, where HNC patients undergo a shortened geriatric assessment as screening, after which an onco-geriatric MDT follows. Based on these two evaluation moments the decision is taken whether to refer the patient for CGA. Using this new two-step care pathway, 30% of the patients were referred to a geriatrician for CGA. This is less compared to other screening tools, therefore reducing the burden for patients and health professionals.

This study identified polypharmacy, age, cognitive deficits and a major treatment intensity as independent variables associated with referral to a geriatrician for CGA. Furthermore, malnutrition was independently associated with for treatment related adverse events, emphasizing the importance of screening for and the treatment of malnutrition. This study offers insights in a new care pathway that helps clinicians to identify the HNC patients who could benefit the most from a CGA by a geriatrician. Each institution, offering care for HNC patients would benefit of the introduction of a comparable care pathway. Referring of frail patients for CGA, based on geriatric screening at the first presentation of the patients, followed by onco-geriatric MDT may help in the decision making, tailored treatment, pre-habilitation and deciding proper perioperative measures for frail patients. Furthermore, appropriate judgement of the biological age may also prevent undertreatment of fit elderly and overtreatment of the frail young ones. Among all geriatric items, malnutrition seems to be most important for adverse outcomes.

### Supplementary Information

Below is the link to the electronic supplementary material.

Supplementary file1 (DOCX 58 KB).

## Data Availability

The data that support the findings are available from the corresponding author upon reasonable request. Data are located in controlled access data storage at the University Medical Center Groningen.
