# Supplementary material for: Evaluation of a new two-step frailty assessment of head and neck patients in a prospective cohort
Source: Eur Arch Otorhinolaryngol. 2024 Apr 23;281(8):4291–304. doi: 10.1007/s00405-024-08651-8 (PMC11266264; doi:10.1007/s00405-024-08651-8)
Supplement: Supplementary file 1 — Supplementary file1 (DOCX 58 KB). [file 405_2024_8651_MOESM1_ESM.docx]

**Supplementary tables**

**Supplementary Table 1** Overview of the tests included in the geriatric screening with corresponding ranges and cut-off values

| Domain | Assessments | Range | Cut-off value |
| --- | --- | --- | --- |
| Somatic | Malnutrition Universal Screening Tool (MUST) | 0-6 | <1: low risk  ≥1:intermediate risk/high risk |
|  | Polypharmacy | 0-∞ | ≥5 |
| Psychological | Delirium Risk | 0-5 | ≥1 |
|  | Mini Mental State Examination (MMSE) | 0-30 | ≤24 |
|  | Patient Health Questionnaire-2 (PHQ-2) | 0-6 | ≥3 |
| Intoxications | Smoking | Not applicable | Not applicable |
|  | Drinking | Not applicable | Not applicable |
| Functional | Katz Activities of Daily Living (ADL) | 0-6 | >1 |
|  | Lawton Instrumental Activities of Daily Living (IADL) | 0-8 | ≤6 |
|  | Mobility (Time get up and go – TUG) | 0-∞ | ≥12 sec |
|  | Fall risk/ Fall in last 6 months | 0-1 | 1 |
| Socioenvironmental | Social network | Not applicable | Not applicable |
|  | Living situation | Not applicable | Not applicable |

**Supplementary Table 2** Association between geriatric assessment instruments, patient, tumour, and treatment characteristics and postoperative complications (univariable and multivariable logistic regression)

| Variable | Total (%)  N (%) | No-complications  N (%) | Complications  N (%) | Univariable analysis^1^ | | Multivariable analysis^2^ | |
| --- | --- | --- | --- | --- | --- | --- | --- |
|  | **n= 130** | **n= 99** | **n= 26** | **p-value** | **Odds ratio (95% CI)** | **p-value** | **Odds ratio (95% CI)** |
| Age  Mean +/- SD(y) | 74.8 ± 9.6 y | 75.1 ± 9.8 y | 72.9 ± 9.3 y | p=0.308 | 0.98(0.93-1.02) | p=0.591 | 1.02(0.96-1.08) |
| Sex  Female  Male  Missing | 35 (26.9%)  95 (73.1%)  0 (0.0%) | 26 (26.3%)  73 (73.7%) | 8 (30.8%)  18 (69.2%) | p=0.646 | 1  0.80(0.31-2.06) |  |  |
| Intoxications  Smoking  Never or former  Current  Missing | 95 (73.1%)  25 (19.2%)  10 (7.7%) | 72 (72.7%)  18 (18.2%) | 18 (69.2%)  7 (26.9%) | p=0.393 | 1  1.56(0.56-4.29) |  |  |
| Drinking  Never or former  Current  Missing | 67 (51.5%)  50 (38.5%)  13 (10.0%) | 50 (50.5%)  38 (38.4%) | 14 (53.8%)  10 (37.0%) | p=0.894 | 1  0.94(0.38-2.35) |  |  |
| Tumour site  Skin  Other  Missing | 46 (35.4%)  72 (55.4%)  12 (9.2%) | 34 (34.3%)  56 (56.6%) | 8 (30.8%)  15 (57.7%) | p=0.791 | 1  1.14(0.44-2.97) |  |  |
| Stage of disease  Early stage (0-II)  Advanced stage (III-IV)  Missing | 60 (46.2%)  67 (51.5%)  3 (2.3%) | 53 (53.5%)  44 (44.4%) | 5 (19.2%)  20 (76.9%) | **p=0.004** | 1  **4.82(1.67-13.88)** |  |  |
| Treatment intensity  Minor (surgery <120 min)  Major (surgery ≥120 min)  Missing | 50 (38.5%)  73 (56.2%)  7 (5.4%) | 45 (45.5%)  49 (49.5%) | 3 (11.5%)  23 (88.5%) | **p=0.003** | 1  **7.04(1.98-25.06)** | **p=0.003** | **1**  **26.55(3.11-226.29)** |
| Referred to geriatrician  No  Yes  Missing | 94 (72.3%)  35 (26.9%)  1 (0.8%) | 74 (74.7%)  24 (24.2%) | 16 (61.5%)  10 (38.5%) | p=0.160 | 1  1.93(0.77-4.81) |  |  |
| Somatic/physic  Polypharmacy  <5 medications  ≥5 medications  Missing | 56 (43.1%)  66 (50.8%)  8 (6.2%) | 43 (43.4%)  50 (50.5%) | 11 (42.3%)  13 (50.0%) | p=0.937 | 1  1.04(0.42-2.55) |  |  |
| MUST  Low risk (<1)  Medium to high risk (≥1)  Missing | 68 (52.3%)  16 (12.3%)  46 (35.4%) | 53 (53.5%)  11 (11.1%) | 12 (46.2%)  3 (11.5%) | p=0.798 | 1  1.21(0.29-4.99) |  |  |
| Psychological  Delirium Risk  Low risk (<1)  High risk (≥1)  Missing | 56 (43.1%)  52 (40.0%)  22 (16.9%) | 38 (38.4%)  47 (47.5%) | 15 (57.7%)  4 (15.4%) | **p=0.011** | 1  **0.22(0.07-0.70)** | **p=0.009** | **1**  **0.18(0.05-0.65)** |
| MMSE  Normal cognition (>24)  Declined cognition (≤24)  Missing | 87 (66.9%)  19 (14.6%)  24 (18.5%) | 67 (67.7%)  16 (16.2%) | 16 (61.5%)  3 (11.5%) | p=0.725 | 1  0.79(0.20-3.02) |  |  |
| PHQ-2  No depression (<3)  Depression (≥3)  Missing | 103 (79.2%)  14 (10.8%)  13 (10.0%) | 77 (77.8%)  11 (11.1%) | 23 (88.5%)  1 (3.8%) | p=0.267 | 1  0.30(0.04-2.48) |  |  |
| Functional  KATZ-ADL  No restrictions (≤1)  Restrictions (>1)  Missing | 95 (73.1%)  13 (10.0%)  22 (16.9%) | 72 (72.7%)  13 (13.1%) | 19 (73.1%)  0 (0.0%) | **Not possible** |  |  |  |
| IADL  No restrictions (>6)  Restrictions (≤6)  Missing | 50 (38.5%)  54 (41.5%)  26 (20.0%) | 40 (40.4%)  41 (41.4%) | 9 (34.6%)  10 (38.5%) | p=0.874 | 1  1.08(0.40-2.95) |  |  |
| TUG  Normal (<12 sec)  Delayed (≥ 12 sec)  Missing | 50 (38.5%)  45 (34.6%)  35 (26.9%) | 37 (37.4%)  37 (37.4%) | 13 (50.0%)  5 (19.2%) | p=0.064 | 1  3.89(0.93-16.32) |  |  |
| Fall risk/History of falls  No  Yes  Missing | 83 (63.8%)  10 (7.7%)  37 (28.5%) | 64 (64.6%)  8 (8.1%) | 15 (57.7%)  2 (7.7%) | p=0.939 | 1  1.07(0.21-5.55) |  |  |
| Social  Living situation  Independent/Independent with help  Institutionalized  Missing | 125 (96.2%)  2 (1.5%)  3 (2.3%) | 79 (79.8%)  17 (17.2%) | 19 (73.1%)  5 (19.2%) | p=0.330 | 1  4.00(0.24-66.29) |  |  |
| Social network  Small  Good  Missing | 16 (12.3%)  80 (61.5%)  34 (26.2%) | 15 (15.2%)  58 (58.6%) | 1 (3.8%)  18 (69.2%) | p=0.150 | 1  0.22(0.03-1.74) |  |  |
| ^1^Variables eligible for the forward and backward selection: age, stage of disease, treatment intensity, delirium risk.  ^2^Variables included after forward and backward selection in the multivariable analysis and with a statistically relevant association | | | | | | | |

**Supplementary Table 3** Association between geriatric assessment instruments, patient, tumour, and treatment characteristics and (chemo)radiotoxicity (univariable and multivariable logistic regression)

| Variable | Total  N (%) | No acute radiation-induced toxicity (grade ≤1)  N (%) | Acute radiation-induced toxicity (grade ≥2)  N (%) | Univariable analysis^1^ | | Multivariable analysis^2^ | |
| --- | --- | --- | --- | --- | --- | --- | --- |
|  | **n= 184** | **n= 79** | **n= 50** | **p-value** | **Odds ratio (95% CI)** | **p-value** | **Odds ratio (95% CI)** |
| Age  Mean +/- SD(y) | 71.7 ± 10.1 y | 72.0 ± 8.4 y | 68.1 ± 9.5 y | **p=0.018** | **0.95(0.91-0.99)** | **p=0.015** | **0.93(0.88-0.99)** |
| Sex  Female  Male  Missing | 50 (27.2%)  134 (72.8%)  0 (0.0%) | 20 (25.3%)  59 (74.7%) | 14 (28.0%)  36 (72.0%) | p=0.736 | 1  0.87(0.39-1.94) |  |  |
| Intoxications  Smoking  Never or former  Current  Missing | 112 (60.9%)  65 (35.3%)  7 (3.8%) | 48 (60.8%)  28 (30.4%) | 26 (52.0%)  23 (46.0%) | p=0.263 | 1  1.52(0.73-3.15) |  |  |
| Drinking  Never or former  Current  Missing | 92 (50.0%)  87 (47.3%)  5 (2.7%) | 38 (48.1%)  39 (49.4%) | 21 (42.0%)  28 (56.0%) | p=0.477 | 1  1.30(0.63-2.67) |  |  |
| Tumour site  Skin  Other  Missing | 32 (17.4%)  144 (78.3%)  8 (4.3%) | 12 (15.2%)  62 (78.5%) | 2 (4.0%)  47 (94.0%) | p=0.055 | 1  4.55(0.97-21.31) |  |  |
| Stage of disease  Early stage (0-II)  Advanced stage (III-IV)  Missing | 44 (23.9%)  139 (75.5%)  1 (0.5%) | 20 (25.3%)  59 (74.7%) | 9 (18.0%)  41 (82.0%) | p=0.334 | 1  1.54(0.64-3.73) |  |  |
| Treatment intensity  Minor (local radiotherapy)  Major (locoregional + regional radiotherapy)  Missing | 58 (31.5%)  121 (65.8%)  5 (2.7%) | 17 (21.5%)  62 (78.5%) | 9 (18.0%)  41 (82.0%) | p=0.628 | 1  1.25(0.51-3.10) |  |  |
| Referred to geriatrician  No  Yes  Missing | 121 (65.8%)  63 (34.2%)  0 (0.0%) | 55 (69.6%)  24 (30.4%) | 34 (68.0%)  16 (32.0%) | p=0.846 | 1  1.08(0.50-2.31) |  |  |
| Somatic/physic  Polypharmacy  <5 medications  ≥5 medications  Missing | 75 (40.8%)  101 (54.9%)  8 (4.3%) | 37 (46.8%)  39 (49.4%) | 21 (42.0%)  26 (52.0%) | p=0.666 | 1  1.18(0.57-2.44) |  |  |
| MUST  Low risk (<1)  Medium to high risk (≥1)  Missing | 89 (48.4%)  31 (16.8%)  64 (34.8%) | 49 (62.0%)  7 (8.9%) | 22 (44.0%)  11 (22.0%) | **p=0.022** | **1**  **3.50(1.20-10.23)** | **p=0.037** | **1**  **3.31(1.08-10.21)** |
| Psychological  Delirium Risk  Low risk (<1)  High risk (≥1)  Missing | 75 (40.8%)  76 (41.3%  33 (17.9%) | 33 (41.8%)  31 (39.3%) | 17 (34.0%)  23 (46.0%) | p=0.369 | 1  1.44(0.65-3.19) |  |  |
| MMSE  Normal cognition (>24)  Declined cognition (≤24)  Missing | 126 (68.5%)  23 (12.5%)  35 (19.0%) | 58 (73.4%)  8 (10.1%) | 33 (66.0%)  5 (10.0%) | p=0.878 | 1  1.10(0.33-3.63) |  |  |
| PHQ-2  No depression (<3)  Depression (≥3)  Missing | 151 (82.1%)  18 (9.8%)  15 (8.2%) | 68 (86.1%)  7 (8.9%) | 43 (86.0%)  5 (10.0%) | p=0.843 | 1  1.13(0.34-3.79) |  |  |
| Functional  KATZ-ADL  No restrictions (≤1)  Restrictions (>1)  Missing | 140 (76.1%)  17 (9.2%)  27 (14.7%) | 66 (83.5%)  2 (2.5%) | 36 (72.0%)  4 (8.0%) | p=0.145 | 1  3.67(0.64-21.00) |  |  |
| IADL  No restrictions (>6)  Restrictions (≤6)  Missing | 77 (41.8%)  70 (38.0%)  37 (20.1%) | 36 (45.6%)  29 (36.7%) | 23 (46.0%)  15 (30.0%) | p=0.611 | 1  0.81(0.36-1.83) |  |  |
| TUG  Normal (<12 sec)  Delayed (≥ 12 sec)  Missing | 122 (66.3%)  15 (8.2%)  47 (25.5%) | 56 (70.9%)  5 (6.3%) | 34 (68.0%)  3 (6.0%) | p=0.988 | 1  0.99(0.22-4.40) |  |  |
| Fall risk/History of falls  No  Yes  Missing | 118 (64.1%)  8 (4.3%)  58 (31.5%) | 57 (72.2%)  1 (1.3%) | 31 (62.0%)  3 (6.0%) | p=0.146 | 1  5.52(0.55-55.30) |  |  |
| Social  Living situation  Independent/Independent with help  Institutionalized  Missing | 175 (95.1%)  8 (4.3%)  1 (0.5%) | 77 (97.5%)  1 (1.3%) | 49 (98.0%)  1 (2.0%) | p=0.751 | 1  1.57(0.10-25.71) |  |  |
| Social network  Small  Good  Missing | 24 (13.0%)  109 (59.2%)  51 (27.7%) | 9 (11.4%)  53 (67.1%) | 9 (18.0%)  27 (54.0%) | p=0.201 | 1  1.96(0.70-5.52) |  |  |
| ^1^Variables eligible for the forward and backward selection: age, MUST.  ^2^Variables included after forward and backward selection in the multivariable analysis and with a statistically relevant association | | | | | | | |
